# Late onset hyperornithinemia, hyperammonemia, and homocitrullinuria syndrome, presenting as recurrent metabolic encephalopathy, A case report

**DOI:** 10.1016/j.amsu.2022.104842

**Published:** 2022-11-08

**Authors:** Fajr M A Sarhan, Afnan W.M. Jobran, Ali Fayyad, Zaid Ghanim, Imad Dweikat, Shireen Elewie, Ala Mustafa Habboub

**Affiliations:** a Al-Quds University-School of Medicine, Abu-Dis, East Jerusalem, Palestine; b Internal Medicine Department, Palestine Medical Complex, Ramallah, West Bank, Palestine; c Neurology Department, Palestine Medical Complex, Ramallah, West Bank, Palestine; d Radiology Department, Palestine Medical Complex, Ramallah, West Bank, Palestine

**Keywords:** Urea-cycle, Hyperornithinemia, Hyperammonemia, Homocitrullinuria, Late-onset, Rare, Biochemistry

## Abstract

**Introduction:**

and importance: Hyperornithinemia, hyperammonemia, and homocitrullinuria (HHH) syndrome (OMIM 238970) seems to be an autosomal recessive disorder caused by a mitochondrial ornithine transporter 1 deficiency, which results in urea cycle dysfunction. HHH is the most uncommon of the urea cycle diseases, with less than 100 cases recorded.

**Case presentation:**

A previously healthy 29 year old male presented to the emergency department complaining of decreased level of consciousness. CT scan, Cerebro-spinal-fluid analysis and toxicology screen were non-significant. Extended serum analysis showed elevated levels of ammonia. Urgent amino acid level analysis showed elevated ornithine. Follow up genetic testing showed that the patient is homozygous for the mutation c.44delG in exon 3 of SLC25A15 gene.

**Clinical discussion:**

In this case, HHH syndrome presented as a late-onset metabolic encephalopathy. For diagnosis; elevated levels of ammonia, ornithine accompanied by the abovementioned genetic mutation confirms the diagnosis. Treatment focuses on reduction of the ammonia levels using sodium benzoat, citrulline or arginine, and low protein diet.

**Conclusions:**

HHH syndrome, which is a urea cycle disorder, can present as a late-onset metabolic encephalopathy. High suspicion for genetic causes of metabolic encephalopathy should be maintained even for older patients without prior diagnosis in childhood/adolescence.

## Highlights

• Less than 100 cases of Hyperornithinemia-Hyperammonemia-Homocitrullinuria (HHH) syndrome has been reported.
• High-index of suspicion for genetic causes for metabolic encephalopathies is to be maintained even in the elderly.
• Sodium benzoat, citrulline or arginine, and a low protein diet are cornerstone treatments for HHH syndrome.

## 1 Introduction

The urea cycle, which converts ammonia to urea, is the most common route for nitrogen disposal. The cycle's operation is dependent on the coordinated activity of five enzymatic stages and membrane transporters, which ensure that the mitochondrial and cytoplasmic components of the cycle are properly integrated. Urea cycle deficiencies can be caused by mutations in either the component enzymes or the transporters, with the clinical severity varying depending on the extent of the functional change. Defects in the urea cycle can occur at any age. Hyperammonemia and clinical symptomatology are frequently elicited by an environmental stressor, usually an infection, overpowering an already impaired urea cycle [1].

## 2 Case presentation

**Fig. 1:**
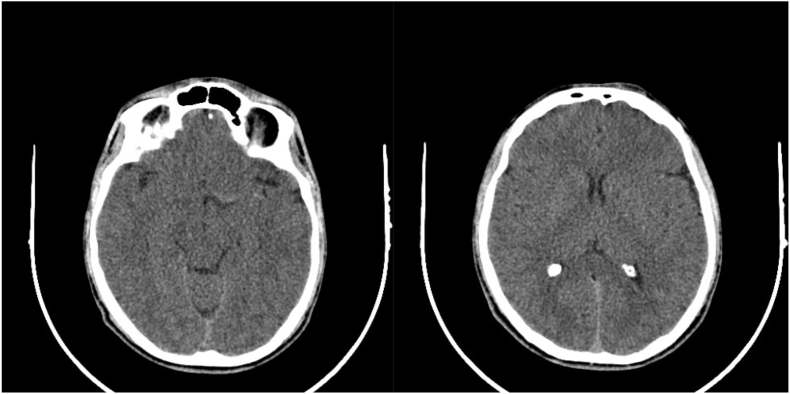
A and B showing normal head CT scans.

A 29-year-old male patient presented to the emergency department via ambulance by his family due to a two-day duration of decreased level of consciousness. The above mentioned patient was in his usual state of health until two weeks ago, when he became confused, less active, and unresponsive. The patient was rushed to the emergency room, where he had an initial CT scan that showed no significant findings (Fig. 1A and B). CSF analysis was normal. Toxicology screening was done and was negative. Physical examination was unremarkable. The patient was discharged home and advised to follow up at the neurology clinic. The family sought alternative medical help at a local “specialist”. The patient's status continued to deteriorate, and gradually became inactive, sleepy, and then became obtunded while showing no response to pain or verbal command. The patient had no history of headaches, fever, jerky body movements, shortness of breath or cough, chest pain or palpitations, recent drug use, no known food or drug allergy. There is a history of similar presentation in his brother that improved spontaneously.

On examination, the patient looks sleepy, responding weakly to pain, dehydrated and not alert. Glasgow Coma Scale was 9/15. Vital signs showed blood pressure of 114/70. Temperature of 37, spo2 97%, HR of 74. The patient's Body Mass Index was 23.4 kg/m2.

**Fig. 2:**
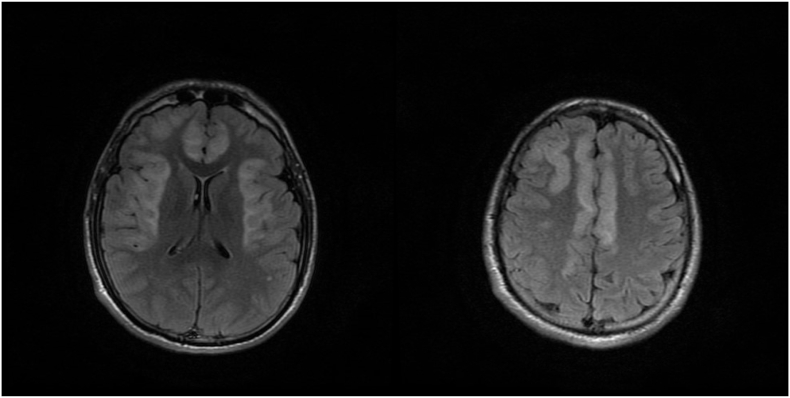
A and B: Axial T2 FLAIR MRI showing hyper intensities in the frontal and temporal lobes.

**Fig. 3:**
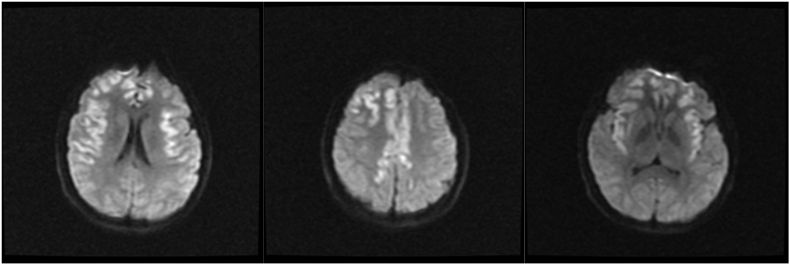
A, B, and 3C: Diffusion Weighted MRI showing signs of acute infarction in the temporal and frontal lobes.

**Fig. 4:**
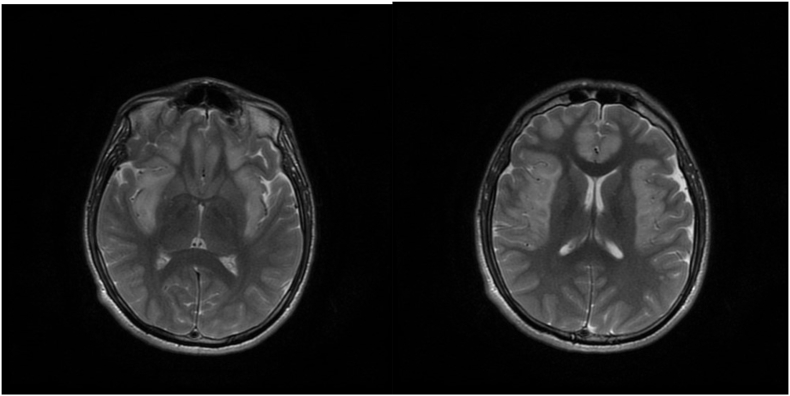
A, B: Fast recovery fast spin echo (FRFSE) MRI

**Fig. 5:**
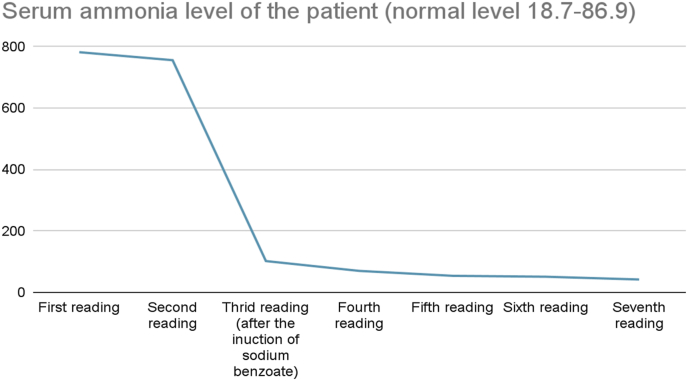
Shows his ammonia level from the day of admission till the discharge.

Power and sensation could not be assessed. His reflexes are brisk, positive Babinski signs bilaterally. The rest of the examination was normal. The patient was admitted. Complete blood count, serum chemistries, and blood urea nitrogen were normal. Repeated CT scan showed no significant findings, CSF analysis was redone and was normal. The decision for a brain MRI was done, and showed diffuse hyper-intensity on flair and T2 (Fig. 2A and B, Fig. 3A, B, and 3C, and Fig. 4A and B) (see Fig. 5).

Extended laboratory testing showed an elevated level of ammonia of 782 (normal level 18.7–86.9). Investigations for hyperammonemia induced encephalopathy were done via the metabolic unit of the hospital. Urgent plasma amino acid levels and urine organic acid were taken, meanwhile the patient was started on sodium benzoate 5 g every 8 hours, arginine 500 mg 5 capsules every 8 hours, and a low protein diet. The patient status had significantly improved, and they decided that the patient can be discharged on the aforementioned medications. Interviewing the patient afterwards, revealed that the patient had recently increased his protein intake for his body-building routine.

Results of plasma amino acid levels showed significantly elevated levels of ornithine 361 μmol/L (normal level 10-163 μmol/L). A diagnosis of HHH syndrome was made. The patient treatment regimen now has citrulline 5 g every 8 hours instead of the arginine, and the patient was advised for genetic testing.

Genetic testing showed that the patient is homozygous for the mutation c.44delG in exon 3 of SLC25A15 gene.

The patient is currently following up at the neurology and metabolic clinics and is showing significant improvement in his daily functioning while maintaining his current medications.

### 2.1 Clinical Discussion

Urea cycle is an essential five-step process in which ammonia is converted to urea at the rate of 10–20 g daily. It's a tightly regulated process that involves both the mitochondrial and the cytoplasmic components on the cell [2,3]. The First step, which is the rate limiting step, happens in the mitochondria, and combines Carbon dioxide and Ammonia to form Carbamoyl Phosphate (CP), which is then sent to the cytoplasm. The second step is the combination of the CP and ornithine to form citrulline. The third step is the conversion of citrulline to argininosuccinate. The fourth step is the splitting of argininosuccinate to arginine and fumarate. The final step is the conversion of arginine to urea and ornithine [2].

Disorders affecting the cycle can lead to the accumulation of ammonia, leading to symptomatic hyperammonemia. Etiologies can be either acquired defects or congenital enzymopathy. Symptomatic hyperammonemia presents as lethargy, confusion, slurred speech, asterixis, convulsions and even coma [2,3].

The pathophysiology of the Central nervous system findings can be due to the cerebral edema and the low levels of glutamate, which works as an excitatory neurotransmitter. Glutamate decreases due to the conversion of glutamate to glutamine, which depresses neuronal activity [3,4].

Hyperornithinemia-Hyperammonemia-Homocitrullinuria (HHH) syndrome or the ornithine translocation deficiency [5] is the rarest autosomal recessive disorder with various genetic mutations on the SLC25A15 (ORNT1 gene), which encodes the mitochondrial ornithine transporter. HHH has been reported in less than 100 cases worldwide [[6], [7], [8], [9]].

The way that HHH presents and the age of onset varies, even with individuals with the same genetic mutation. The neonatal onset of HHH accounts for 8%, while other ages account for the rest. The non-neonatal presentation causes a spectrum of manifestations ranging from acute hyperammonemic encephalopathy, chronic neurocognitive symptoms, and/or chronic liver dysfunction [[10], [11], [12]]. The ChNS symptoms are due to the progressive pyramidal and cerebellar dysfunction [11]. Most cases are presented by childhood, in symptoms which are learning disabilities, cognitive changes, spasticity and liver dysfunction myoclonus epilepsy [5]. Adulthood presentation without prior neurological symptoms is rarely encountered [10,12,13].

Diagnosis is divided into biochemical testing, which is dependent on the episodic or postprandial hyperammonemia, persistent hyperornithinemia, and urinary Homocitrullinuria, additional testing will show coagulopathy, and elevation in the liver enzymes [13]; while the genetic testing detects the mutations on the SLC25A15 gene [[10], [11], [12]]. Kamer Tezcan et al. confirmed that the reason why later onset disorders happen comes from the fact that other ornithine transporters are found on the mitochondria which includes ORNT2 (SLC25A2) and, more recently, ORNT3 (SLC25A29). Those transporters cause the delay from the neonatal presentation [13].

Management is split into acute and chronic phases. While the acute phase focuses on stopping protein intake for 24 hours and administering glucose [14]. Low doses of arginine is to be maintained to replace the intermediates of the urea cycle [14,15]. Ammonia scavengers such as sodium benzoate are to be used in both acute and chronic phases. Chronic phase management depends on the low protein diet, with arginine supplementation [[14], [15], [16]]. This case report has been reported in line with the SCARE Criteria [17].

## 3 Conclusions and take home messages

This case illustrates a late-onset HHH syndrome, which is a very rare urea cycle disorder. It can present as an early onset or a late-onset metabolic encephalopathy. High suspicion for genetic causes of metabolic encephalopathy should be maintained even for older patients without prior diagnosis in childhood/adolescence. Sodium benzoat, citrulline or arginine, and low protein diet are cornerstone treatments for HHH syndrome.

## Ethical approval

This study is exempt from ethical approval in out institution.

## Sources of funding

The authors declare that writing and publishing this manuscript was not funded by any organization.

## Author contribution

Writing the manuscript: Fajr M A Sarhan, Afnan W.M. Jobran, Ali Fayyad, Zaid Ghanim, Imad Dweikat, Ala Mustafa Habboub.

Imaging description: Ala Mustafa Habboub, Zaid Ghanim, Fajr M A Sarhan, Shireen Elewie.

Reviewing & editing the manuscript: Imad Dweikat, Fajr M A Sarhan, Ali Fayyad, Afnan W.M. Jobran, Ala Mustafa Habboub.

## Consent

A written informed consent for the data and picture was taken from the patient and the family and available upon request from the Editor-in-Chief.

## Registration of research studies

Not Applicable.

## Guarantor

Fajr M A Sarhan.

## Provenance and peer review

Not commissioned, externally peer-reviewed.

## Declaration of competing interest

The authors declare that there is no conflict of interest regarding the publication of this article.
